# Videothoracoscopic surgical approach for spontaneous pneumothorax: review of the pertinent literature

**DOI:** 10.1186/1749-7922-3-23

**Published:** 2008-07-21

**Authors:** Hiroyuki Sakurai

**Affiliations:** 1 Department of Thoracic Surgery, Saiseikai Central Hospital, Tokyo, Japan

## Abstract

Spontaneous pneumothorax is usually caused by the rupture of subpleural blebs/bullae in the underlying lung and is one of the most common elective applications of video-assisted thoracoscopic surgery (VATS). VATS has been used as an alternative to thoracotomy in the treatment of spontaneous pneumothorax. Recurrent pneumothorax and persistent air leakage are quite often indications for spontaneous pneumothorax, and bilateral spontaneous pneumothorax is also considered to be an indication for surgical intervention. The goals of surgical intervention are to eliminate intrapleural air collection and prevent recurrence. Diverse procedures have been reported in the surgical treatment for spontaneous pneumothorax. We review the literature regarding the VATS approach for spontaneous pneumothorax.

## Background

With advances in videothoracoscopic equipment, most operations for spontaneous pneumothorax are usually performed with a minimally invasive technique that involves video-assisted thoracoscopic surgery (VATS) [1]. Additionally, VATS has the advantage that inspection of the pleura and lung is more complete than when carried out through limited thoracotomy. This VATS procedure is generally done in the full lateral decubitus position under general anesthesia [2-4]. Many researchers have reported a variety of techniques for the treatment of spontaneous pneumothorax via VATS. In this report we review the literature on the VATS approach for spontaneous pneumothorax.

## Treatment of spontaneous pneumothorax

Patients with spontaneous pneumothorax, which is usually caused by a rupture of a subpleural bleb or bulla in the underlying lung, most commonly present with ipsilateral sudden chest pain and dyspnea. Spontaneous pneumothoraces are classified as primary or secondary [5-7]. Primary spontaneous pneumothorax occurs in otherwise healthy patients, most commonly in tall, young, lean males and particularly in those who smoke [7]. Secondary spontaneous pneumothorax occurs as a complication of an underlying lung disease, which most often is chronic obstructive pulmonary disease [5,7]. In either case, the goals of pneumothorax treatment are to eliminate intrapleural air collection and to prevent recurrence. In the first episodes of spontaneous pneumothorax, observation, simple aspiration, or chest tube drainage in proportion to clinical stability or the degree of pneumothorax are recommended as first-line therapies [6-12]. The recurrence rate after these therapies has been reported to range from 16% to 52%, with a mean of 30% [5,10,12,13]. Treatment to prevent recurrent pneumothorax is essential under clinical conditions such as spontaneous pneumothorax with potential respiratory insufficiency, recurrent spontaneous pneumothorax, or persistent pneumothorax. Under these conditions, a surgical option is considered for pneumothorax.

## Surgical indications and procedure for spontaneous pneumothorax

The generally accepted indications for surgical intervention in pneumothorax are as follows [6,7,9,13-15]: 1) recurrent ipsilateral pneumothorax, 2) first contralateral pneumothorax, 3) bilateral simultaneous pneumothorax, 4) spontaneous hemopneumothorax, and 5) professions at risk (e.g., pilots, divers). Recently, based on the minimal invasiveness of VATS, several studies have recommended surgical treatment for the first episode of pneumothorax [16-19]. Surgical management aims at the resection of blebs or the suturing of pulmonary perforation and the creation of pleurodesis. The operation is usually performed under general anesthesia and one-lung ventilation. At one time, the surgical approach had been through either standard posterolateral thoracotomy or, more frequently, smaller incisions [15]. In the 1990s, due to the advent of video-imaging technology and instruments, a thoracoscopic procedure, called VATS, became widespread [7]. Since then, most reports on the surgical treatment of spontaneous pneumothorax have dealt with VATS instead of open thoracotomy. In the VATS procedure, patients are usually in the lateral decubitus position, and three ports are generally necessary: one for the thoracoscope and two for the lung graspers and stapling devices [20-22]. Combined stapled resection of blebs and bullae and the creation of pleurodesis (e.g. abrasion, apical pleurectomy, and chemical, laser, or electrocautery pleurodesis) is most commonly performed [7,21-31]. Recently, it was reported that recurrence was prevented by bullectomy and reinforcement of the visceral pleura (covering the stapled line with absorbable mesh) [32]. Although procedures other than stapled resection, including clipping pedicled bullae, ligation or looping of bullous lesions and electrocautery or laser abrasion of small blebs and bullae, have also been described [33,34], they have been reported to be inferior to the stapled resection of blebs and bullae with regard to recurrence. Global recurrence rates after bullectomy accompanied by pleurodesis range from 0 to 10%, and are mostly less than 5% [5,22,25-28,32,35-38]. The number of blebs is associated with the recurrence rate, according to the report by Naunheim et al. [39]. The postoperative recurrence rate was 27.3% when no blebs were indentified, and 0% and 2.7% with one or multiple blebs, respectively [39]. Apical lung wedge resection offers lower recurrence rates even if no blebs (no thoracoscopic abnormalities) are seen [40,41]. On the other hand, if surgical management involves a pleural procedure alone or surgical resection of blebs alone, either is associated with higher recurrence rates (6.3% to 16.0%) [32,36]. In addition, the combination of thoracoscopic bullectomy and chemical pleurodesis, such as with tetracycline, instead of abrasion/pleurectomy is also less successful, except for talc poudrage [15,42]. Vanderschueren's classification [43] is often used to determine the extent of morphologic lung alteration for spontaneous pneumothorax. This classification consists of four different stages: stage I: no endoscopic abnormalities (normal findings), stage II: pleuropulmonary adhesions, stage III: blebs/bullae less than 2 cm in diameter, and stage IV: bullae more than 2 cm in diameter. Different results have been published based on these stages [29]. Stage I pneumothorax is most apt to recur, although the pathophysiology and mechanisms of spontaneous pneumothorax are not well known. In stage I cases, apical lung resection and apical pleurectomy are not sufficient and additional treatment such as talc poudrage might be indicated [29,41].

## Thoracotomy versus VATS

Randomized clinical trials that have compared the clinical results of a VATS procedure to thoracotomy for spontaneous pneumothorax have shown that the VATS procedure was superior to thoracotomy with regard to postoperative pain, hospital stay, and pulmonary function [44-46]. VATS showed no advantage over thoracotomy with regard to the postoperative recurrence rate [15,47]. Based on previous retrospective reports, patients who underwent VATS seemed to be more likely to develop recurrence [5,15,35].

## New technology – needle VATS

According to the recent development of technology in the videoscopic field, several reports have shown that needlescopic VATS is feasible in the treatment of spontaneous pneumothorax [48,49]. The use of needlescopic instruments (2 mm in diameter) has been valuable for further reducing postoperative neuralgia and for wound-healing without a scar.

## Simultaneous bilateral spontaneous pneumothorax

Simultaneous bilateral spontaneous pneumothorax is an infrequent phenomenon that reportedly accounts for approximately 1% of all cases of spontaneous pneumothorax [50,51]. Although the clinical presentation of simultaneous bilateral spontaneous pneumothorax is diverse, it can deteriorate into death [52-55]. First, chest drainage is indispensable in the treatment of bilateral spontaneous pneumothorax. Next, we must consider the need for further treatment, including surgery or pleurodesis [51,53,56,57]. It has been reported that the recurrence rate of pneumothoraces after surgical intervention is far lower than that after medical pleurodesis [5,7]. Therefore, single-stage surgical treatment for bilateral pneumothorax is often selected. Lee and coauthors reported that a lower body mass index and the presence of bilateral blebs/bullae on high-resolution CT were significant independent risk factors for simultaneous bilateral primary spontaneous pneumothorax [58]. Huang et al. reported similar findings and supported the notion that patients with spontaneous bilateral pneumothorax required cautious treatment [59]. On the other hand, based on the fact that approximately 70% of patients with simultaneous bilateral pneumothorax have underlying lung disease [50,56], rather than bullous disease or emphysema, we should also investigate the possible presence of a serious lung disorder such as malignancy, interstitial disease, or tuberculosis [55,56].

## Surgical approach to both lungs

Median sternotomy or bilateral axillary thoracotomy has been traditionally performed for the bilateral treatment of pneumothorax [3,60]. With the advent of VATS, there have been several reports of a bilateral VATS procedure at a single sitting, with excellent results [2,61,62]. The bilateral VATS procedure is usually performed in the full lateral decubitus position [61]. After the first side is complete, the patient is rotated to the opposite lateral decubitus position, and an identical procedure is performed on the contralateral side. As described in several reports on bilateral spontaneous pneumothorax or volume reduction surgery [1,3,4,20,62], the bilateral VATS procedure has also been performed in the supine position. This procedure is useful in several aspects, compared to that in the bilateral decubitus position. With the supine position, specifically, we are not concerned with the amount of labor involved in changing the lateral decubitus position of the patient, kinking of the chest tube during rotation of the patient to the opposite lateral decubitus position, problems in reconfirming placement of the endotracheal tube (double-lumen tube or bronchial blocker), or handling as a cause of iatrogenic pneumothorax of the opposite side during the operation on the first side in a situation without chest tube drainage. When we perform the VATS procedure in the lateral decubitus position, the working ports are usually positioned anteriorly from the tip of the scapula. In the supine procedure, the anterior portion from the tip of the scapula can be included in the operative field. Therefore, as in the lateral decubitus position, bilateral VATS in the supine position can be performed without difficulty by tilting the operative table, especially with regard to an approach to apical and upper mediastinal sites of the lung. If resection is planned in the lower lobes or posteriorly, it may be difficult to achieve satisfactory manipulation of VATS in the supine position [62] because of the elevated arms. A simple bullectomy does not account for all instances of the bilateral VATS procedure in the supine position, and bilateral volume reduction surgery for diffuse pulmonary emphysema in this position can be performed [62]. This VATS procedure in the supine position may be useful for single-stage treatment of bilateral thoracic disease, especially localized in the upper and/or middle lobes of the lung [62]. It saves the trouble of changing the position of the patient and can be efficiently performed by simply tilting the operation table. This supine approach would not be associated with increased morbidity or a prolonged hospital stay compared to staged bilateral VATS [63].

Recently, a few researchers [64-66] have introduced, as a further advanced novel procedure, a thoracoscopic ipsilateral transmediastinal approach to contralateral as well as ipsilateral bullous lesions in patients with bilateral pneumothorax in the supine position, although the results were obtained under limited situations. Further new techniques with the VATS procedure will likely be developed in the future.

## Competing interests

The author declares that they have no competing interests.

## Authors' contributions

The author performed a literature search, and wrote and approved the final manuscript.
